# Synthesis of a Novel Carbocyclic Analog of Bredinin

**DOI:** 10.3390/molecules180911576

**Published:** 2013-09-17

**Authors:** Vasu Nair, Fan Zhang

**Affiliations:** Center for Drug Discovery and College of Pharmacy, University of Georgia, Athens, GA 30602, USA

**Keywords:** bredinin, carbocyclic analog, synthesis, intramolecular cyclization, antiviral activity

## Abstract

The natural nucleoside antibiotic, bredinin, exhibits antiviral and other biological activities. While various nucleosides related to bredinin have been synthesized, its carbocyclic analog has remained unknown. Synthesis of this heretofore unknown analog of bredinin is described. The key precursor, (3a*S*,4*R*,6*R*,6a*R*)-6-((methoxy-methoxy)methyl)-2,2-dimethyltetrahydro-3a*H*-cyclopenta[*d*][1,3]dioxol-4-amine (**5**), was prepared from the commercially available compound, (1*R*,4*S*)-2-azabicyclo[2.2.1] hept-5-en-3-one (**4**). Our initial approach used intermediate **6**, derived in three transformations from **5**, for the key photolytic step to produce the desired ring-opened precursor to the target compound. This photochemical transformation was unsuccessful. However, an appropriately protected and related precursor was synthesized from 5 through the following side-chain functional group transformations: elaboration of the amino group through malonyl ester formation, oximation at the central carbon, conversion of ester to amide and catalytic reduction of the oxime group. This precursor, on treatment with triethylorthoformate and catalytic acetic acid in ethanol, underwent cyclization to produce the desired 4-carbamoyl-imidazolium-5-olate ring. Deprotection of the latter product proceeded smoothly to give the carbocyclic analog of bredinin. This target molecule exhibits antiviral activity, albeit low, against a number of RNA viruses. Further biological evaluations are in progress.

## 1. Introduction

**Figure 1:**
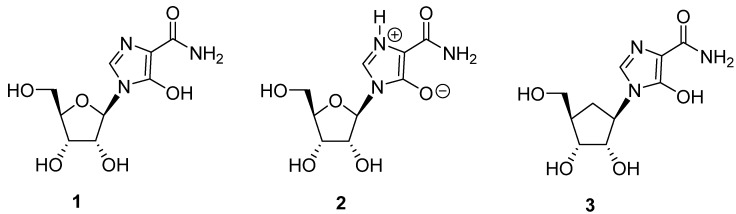
Structure of bredinin 1, its zwitterionic form 2 [6] and its carbocyclic analog 3.

Bredinin (**1**; zwitterion form **2**, Figure 1) is an imidazole nucleoside antibiotic, which was originally isolated from the culture filtrates of *Eupenicillium brefeldianum* [1]. This natural nucleoside, also called mizoribine, exhibits broad-spectrum antiviral activity, including activity against three strains of respiratory syncytial virus (RSV), one strain each of Flu A and B, parainfluenza virus types 2 and 3, mumps virus, and the measles virus (MLSV) [2]. EC_50_ values in the submicromolar range were observed with some of these viruses. This natural compound also exhibits a synergistic effect against the bovine viral diarrhea virus (BVDV) in combination with IFN-α. The EC_50_ against BVDV in MDBK cells for a 10:1 combination of IFN-α and bredinin was 0.66 μM, whereas bredinin alone showed an EC_50_ of 5.3 μM [3]. Bredinin has also been found to have activity against the hepatitis C virus [4,5].

This compound also shows activity against the vaccinia virus [1] and the human cytomegalovirus, HCMV [7]. It has been approved in Japan for use in the prevention of organ allograft rejection, and in the treatment of rheumatoid arthritis, primary nephrosis, lupus nephritis, dermatomyositis and autoimmune dermatoses [8]. It is also clinically used as an immunosuppressant in renal transplantation [9,10] and for autoimmune diseases such as rheumatism [11].

**Scheme 1:**
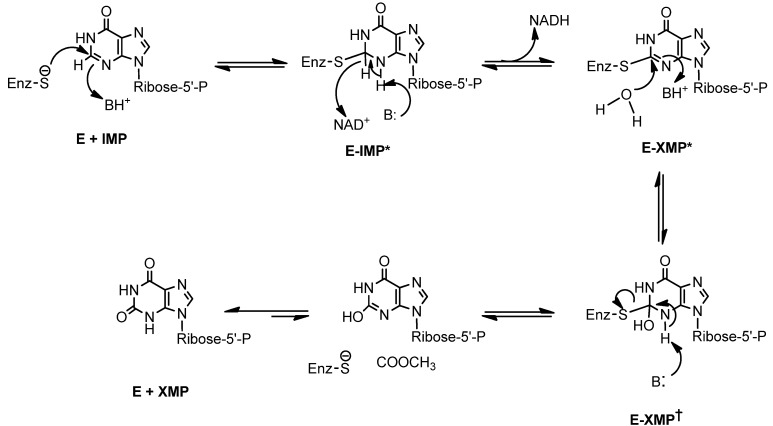
Transition state in the conversion of IMP to XMP catalyzed by IMPDH [12].

Bredinin is one of the most potent known inhibitors of IMPDH (*K_i_* 0.4 nM *E. coli* IMPDH and 4.0 nM for human type II IMPDH). Its active metabolite, bredinin 5'-monophosphate, is thought to mimic the transition state of IMPDH catalysis [12]. Thus, the mechanism of its antiviral activity appears to be associated with inhibition of IMPDH by bredinin monophosphate [12,13] (Scheme 1). Consistent with this suggestion is the observation that the activities of bredinin against viruses can be reversed by guanosine and guanosine monophosphate [2].

Because of the significant biological activity exhibited by bredinin, we sought to develop synthetic routes to carbocyclic analogs of this compound that would be of potential interest in the RNA antiviral drug discovery area. However, an issue with the structure of bredinin is that it exists in the charged zwitterionic form **2** and this has been confirmed by X-ray crystallographic studies [6,14]. The zwitterionic structure of the heterocyclic ring of bredinin has produced significant limitations in the synthesis of new analogs and homologs of this compound. In this report, we provide a methodology for the synthesis of 1-((1*R*,2*S*,3*R*,4*R*)-2,3-dihydroxy-4-(hydroxymethyl)cyclopentyl)-5-hydroxy-1*H*-imidazole-4-carboxamide (**3**), the heretofore unknown carbocyclic analog of bredinin. The methodology described should have generality for the synthesis of other carbocyclic analogs and derivatives of this biologically interesting compound [15,16,17,18,19].

## 2. Results and Discussion

In planning the synthesis of **3** and from the point of view of selectivity, we considered it preferable for this carbocyclic nucleoside to be synthesized through a linear rather than a convergent approach in order to avoid side products resulting from alkylation of the nitrogens of imidazole base and to circumvent possible complications arising from the presence of the hydroxyl group of the imidazole base.

**Scheme 2:**
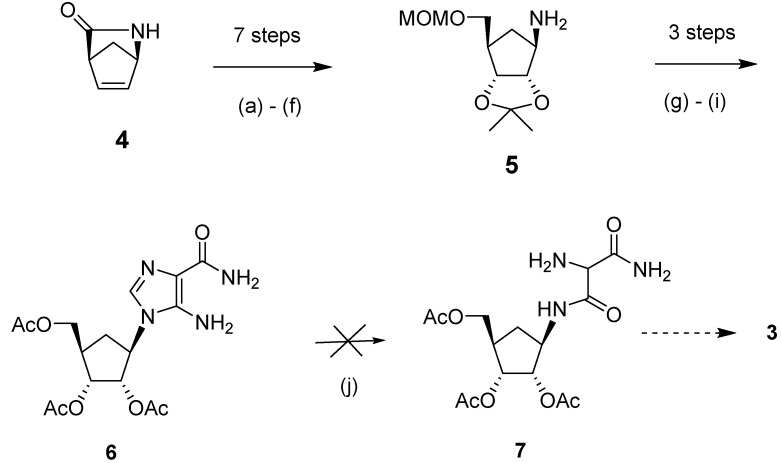
The photochemical approach to the carbocylic analog of bredinin.

We initiated our synthetic effort (Scheme 2) with the commercially available bicyclic lactam, (1*R*,4*S*)-2-azabicyclo[2.2.1] hept-5-en-3-one (**4**). This chiral building block was used because of its versatility in the synthesis of other carbocyclic nucleosides [20]. Synthesis of the key intermediate, (3a*S*,4*R*,6*R*,6a*R*)-6-((methoxymethoxy)methyl)-2,2-dimethyltetrahydro-3a*H*-cyclo-penta[*d*][1,3]dioxol-4-amine (**5**) from **4** involved the following transformations: hydroxylation of **4** through treatment with osmium tetroxide in the presence of *N*-methylmorpholine-*N*-oxide (NMO), which resulted in hydroxylation from the α-face, as previously reported [16,21,22,23]; protection of the diol and the lactam NH groups; reductive cleavage of the lactam and protection of the resulting alcohol; and selective aqueous hydrolysis of the NH protecting group [16]. It is relevant to note that the use of *tert*-butyldimethylsilyl ether (TBDMS) for protection of the 5'-hydroxyl group proved less successful, as the eventual deprotection step became problematic because of the poor water solubility of the TBDMS-protected compound.

*Reagents and conditions:* (**a**) OsO_4_, NMO, H_2_O:t-BuOH (1:1.5), 70 °C, 30 min, 94%; (**b**) 2,2-DMP, TsOH, DMF, r.t., 20 h, 84%; (**c**) Boc_2_O, DMAP, CH_3_CN, r.t., 15 h, 98%; (**d**) NaBH_4_, CH_3_OH, 0 °C, 2 h; (**e**) ClCH_2_OCH_3_, (iPr)_2_NC_2_H_5_, CH_2_Cl_2_, r.t., 5 h; (**f**) water, reflux, 6 h, 94%; (**g**) EtO-CH=NCH(CN)CONH_2_, CH_3_CN, 90 °C, 15 min, 63%; (**h**) 1N HCl, reflux, 1 h, 78%; (**i**) Ac_2_O, (C_2_H_5_)_3_N, DMAP, CH_3_CN, r.t.,1 h, 65%; (**j**) 0.1N HCl, 250 nm or 300 nm UV, r.t., 1 h.

Based on previous literature methodology on the synthesis of bredinin [24,25], we considered intermediate **7** (Scheme 1) to be an appropriate precursor to prepare our target compound **3**, as aminomalonamide derivatives of riboside were reported to cyclize to 5-hydroxyimidazole compounds through treatment with triethyl orthoformate*.* Thus, in order to synthesize **7**, compound **5** was first treated with ethyl-*N*-(carbamoylcyanomethyl)formimidate followed by deprotection of the MOM group and acetylation to produce key precursor **6** (31% yield for three steps). However, the photochemical reaction of **6** with ultraviolet light at λ_max_ 254 nm or 300 nm produced an intractable mixture of products, from which compound **7** could not be detected or isolated. Interestingly, in this photolysis step, carbocyclic intermediate **6** behaved totally differently compared to its ribosyl counterpart [24]. 

**Scheme 3:**
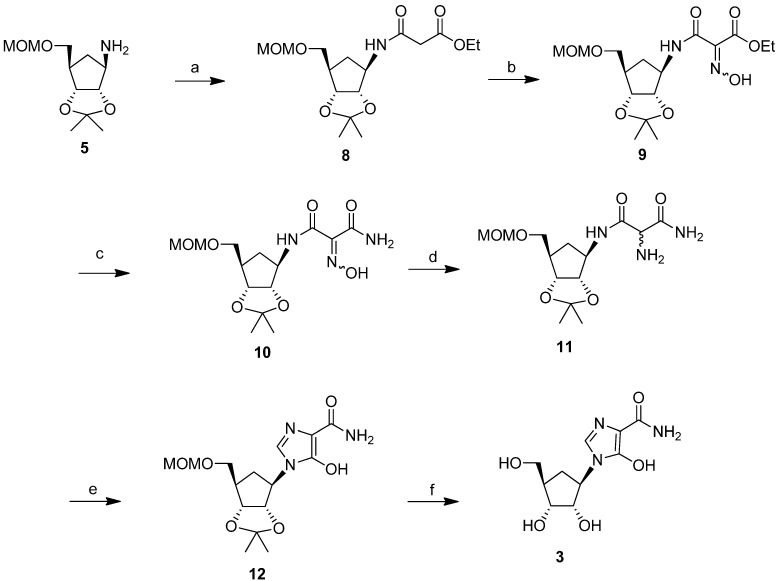
Successful route for the synthesis of a carbocyclic analog of bredinin.

Failure of the photolytic ring opening of imidazole **6** prompted us to investigate alternative pathways [26] to produce intermediate **7** or its derivatives, which could be cyclized to the target compound **3**. The successful strategy that we examined (Scheme 3) was through the synthesis of the malondiamide intermediate **11**. Although this pathway was somewhat longer than the photolytic pathway of Scheme 1, we chose to use this more viable approach to prepare intermediate **11**, in order to circumvent the decomposition problem of the precursor that had been observed during ultraviolet irradiation in the photolysis approach. In this thermal approach, the protected cyclopentylamine **5** was first treated with ethyl 3-chloro-3-oxopropionate in triethylamine to give the amide **8** (73%). Modification of the malonate side chain of **8** by treatment with aqueous sodium nitrite in acetic acid produced the oxime **9** (46%). On treatment with methanolic ammonia at 0 °C for 12 h, oxime **9** was almost quantitatively converted to the malonamide **10**. Reduction of **10** with 10% platinum on carbon gave the desired amine precursor **11** in 77% purified yield.

Most of the reported conditions for cyclisation to produce the imidazole ring in the reported synthesis of bredinin involved heating the malonamide intermediates with orthoformate or formimidate derivatives in DMF [24,27]. These procedures were completely unsuccessful with intermediate **11**. However, cyclization to the 5-hydroxyimidazole ring did occur effectively when malonamide **11** was heated at 90 °C with triethyl orthoformate in the presence of catalytic amounts of acetic acid in ethanol. Purification by HPLC afforded the protected carbocyclic bredinin **12** as a pure product in 55% yield. Deprotection of **12** in methanol under reflux conditions in the presence of 1N HCl gave the target molecule **3** in 54% isolated yield, after HPLC purification (Scheme 3).

*Reagents and conditions:* (**a**) ClCOCH_2_CO_2_C_2_H_5_, (C_2_H_5_)_3_N, CH_3_CN, 0 °C, 30 min, then r.t., 2 h, 73.3%; (**b**) NaNO_2_, CH_3_CO_2_H, THF, r.t., 40 h, 46%; (**c**) NH_3_, CH_3_OH, 0 °C, 12 h, 99.1%; (**d**) H_2_, 10% Pt/C, CH_3_OH, r.t., 0.5 h, 77%; (**e**) HC(OC_2_H_5_)_3_, catalytic CH_3_CO_2_H, C_2_H_5_OH, 90 °C, 1.5 h, 55%; (**f**) 1 N HCl, CH_3_OH, reflux, 1h, 53.6%.

Compound **3** was evaluated for *in vitro* antiviral activity against a number of RNA viruses [28]. However, only low activity was seen with West Nile virus (New York), Rift Valley Fever virus (MP-12), FluA (H1N1) and FluA (H5N1), and Tacaribe virus (TRVL). Further antiviral studies against other RNA viruses are in progress and those results will be reported elsewhere when the studies are completed. 

## 3. Experimental

### 3.1. General Procedures

All commercial chemicals and solvents used in the synthetic steps were purchased from Aldrich or Fisher Scientific and used as received. Reactions were monitored by thin layer chromatography (TLC) using appropriate developing solvents and pre-coated silica gel plates (UV 254 nm) purchased from Merck and Co. Each synthetic reaction was performed under an inert atmosphere of dry nitrogen unless stated otherwise. ^1^H-NMR and ^13^C-NMR spectra were recorded on a Varian Unity Inova 500 MHz or a Varian Mercury Plus 400 MHz NMR spectrometer. Chemical shifts are reported in δ (ppm) relative to tetramethylsilane (TMS) as internal standard and multiplicities are given as singlet (s), doublet (d), quartet (q), multiplet (m) and broad singlet (br s). High resolution mass spectral data were obtained on a Q-TOF Ion Mobility mass spectrometer. Ultraviolet spectra were recorded on Varian Cary Model 3 spectrophotometer. HPLC purifications were performed on an analytical Delta Pak C_18_ column using a Beckman Coulter Gold 127 HPLC system with a Gold 166 UV analytical detector. The data were collected and processed using a Gold software package.

*(1S,2R,3R,5R)-3-(acetoxymethyl)-5-(5-amino-4-carbamoyl-1H-imidazol-1-yl)cyclopentane- 1,2-diyl diacetate* (**6**). To a solution of ethyl N-[ethoxycarbonyl (cyano)methyl] formimidate (0.516 g, 2.80 mmol) in acetonitrile (5 mL) was added a solution of amine **5** [16,23] (0.6 g, 1.81 mmol) in acetonitrile (10 mL) and the mixture was refluxed at 90 °C for 15 min. The reaction mixture was concentrated to dryness, and the residue was purified by silica gel column chromatography using chloroform/methanol (98:2) as eluent to give the imidazole intermediate (0.423 g, 63.3%). ^1^H-NMR (500 MHz, CDCl_3_) δ 7.00 (s, 1H), 6.48 (bs, 1H), 5.60 (brs, 2H), 4.69–4.67 (m, 2H), 4.60–4.58 (m, 1H), 4.43 (t, *J* = 7.0 Hz, 1H), 4.26–4.21 (m, 1H), 3.73–3.71 (m, 1H), 3.67–3.64 (m, 1H), 3.40 (s, 3H) 3.34 (m, 1H), 2.55–2.50 (m, 1H), 2.47–2.42 (m, 1H), 2.26–2.19 (m, 1H), 1.61 (s, 3H), 1.31 (s, 3H). ^13^C-NMR (125 MHz, CDCl_3_) δ 167.1, 150.0, 144.1, 126.4, 114.6, 113.6, 96.7, 85.5, 81.4, 67.9, 60.6, 55.5, 43.2, 31.6, 27.3, 24.9. This product (0.1 g, 0.29 mmol) was deprotected by heating at 90 °C in 1N hydrochloric acid (10 mL) for 1 h. After cooling to room temperature, the reaction mixture was neutralized with a saturated solution of sodium bicarbonate (50 mL). The solvent was removed under reduced pressure, and the residue was purified by HPLC (C_18_ column) to give the deprotected product (0.058 g, 78.0%). ^1^H NMR (500 MHz, D_2_O) δ 7.35 (s, 1H), 4.26–4.21 (m, 1H), 4.12–4.09 (m, 1H), 3.89–3.87 (m, 1H), 3.55 (d, *J* = 6.0 Hz, 2H), 2.35–2.29 (m, 1H), 2.12–2.09 (m, 1H), 1.51–1.45 (m, 1H). Acetic anhydride (0.094 mL, 1.0 mmol) was added dropwise to a mixture of the deprotected compound (0.052 g, 0.2 mmol), triethylamine (0.27 mL, 2.0 mmol) and DMAP (2 mg, 0.0164 mmol) in acetonitrile (5 mL). The reaction mixture was stirred for 1 h at room temperature and MeOH (0.5 mL) was added to decompose the excess acetic anhydride. The solution was concentrated under vacuum and the residue was purified by silica gel column chromatography to give 6 (0.050 g, 65.1%). ^1^H-NMR (500 MHz, CDCl_3_) δ 7.16 (s, 1H), 6.61 (brs, 1H), 5.37 (brs, 1H), 5.23 (s, 2H), 5.20-5.18 (m, 1H), 5.16–5.13 (m, 1H), 4.50–4.45 (m, 1H), 4.28–4.24 (m, 1H), 4.19–4.14 (m, 1H), 2.63–2.53 (m, 2H), 2.12 (s, 3H), 2.09 (s, 3H), 2.06 (s, 3H), 1.95–1.90 (m, 1H). ^13^C-NMR (125 MHz, CDCl_3_) δ 170.8, 170.1, 169.9, 166.9, 143.1, 126.9, 114.5, 76.3, 71.8, 63.9, 55.4, 40.0, 28.0, 20.9, 20.8, 20.7.

*Ethyl 3-((3aS,4R,6R,6aR)-6-((methoxymethoxy)methyl)-2,2-dimethyltetrahydro-3aH-cyclopenta[d][1,3]-dioxol-4-yl-amino)-3-oxopropanoate* (**8**). To a solution of **5** (0.349 g, 1.50 mmol), triethylamine (0. 607 g, 6.00 mmol) in acetonitrile (5 mL) was added ethyl 3-chloro-3-oxopropionate (0.248 g, 1.65 mmol) at 0 °C. This reaction mixture was stirred at 0 °C for 30 min and then at room temperature for 2 h. The solvent was removed under reduced pressure and the residue was purified by silica gel column chromatography using chloroform/methanol (99:1) to give compound **8** (0.38 g, 73.3%). ^1^H-NMR (500 MHz, CDCl_3_) δ 7.64 (d, *J* = 8.0 Hz, 1H), 4.69 (d, *J* = 7.0 Hz, 1H), 4.67 (d, *J* = 7.0 Hz, 1H), 4.52–4.50 (m, 1H), 4.41–4.39 (m, 1H), 4.37–4.36 (m, 1H), 4.17 (q, *J* = 7.0 Hz, 2H), 3.66–3.64 (m, 1H), 3.56–3.53 (m, 1H), 3.37 (s, 3H), 3.27 (s, 2H), 2.51–2.47 (m, 1H), 2.40–2.38 (m, 1H), 1.53–1.49 (m, 1H), 1.47 (s, 3H), 1.27 (t, *J* = 7.0 Hz, 3H), 1.27 (s, 3H); ^13^C-NMR (125 MHz, CDCl_3_) δ 169.4, 164.4, 111.5, 96.6, 86.7, 83.5, 69.3, 61.5, 55.7, 55.4, 45.1, 41.5, 33.4, 27.1, 24.7, 14.0; HRMS (ESI) calcd for C_16_H_28_NO_7_:[M+H]^+^ = 346.1866, found 346.1867.

*Ethyl 2-(hydroxyimino)-3-((3aS,4R,6R,6aR)-6-((methoxymethoxy)methyl)-2,2-dimethyltetrahydro-3aH-cyclopenta[d][1,3]dioxol-4-ylamino)-3-oxopropanoate* (**9**). A solution of sodium nitrite (1.1 g, 16 mmol) in H_2_O (1 mL) was added to compound **8** (0.360 g, 1.04 mmol) in acetic acid (4 mL) and tetrahydrofuran (4 mL) and the reaction mixture was stirred at room temperature for 40 h and then extracted with ethyl acetate (2 × 20 mL). The combined organic extracts were dried (Na_2_SO_4_) and concentrated under vacuum to give a yellow oil which was purified by silica gel column chromatography using CHCl_3_/CH_3_OH (98:2) to give **9** (0.18 g, 46%). ^1^H-NMR (500 MHz, CDCl_3_) δ 10.3 (brs, 1H), 7.96 (d, *J* = 9.0 Hz, 1H), 4.75–7.72 (m, 2H), 4.60–4.58 (m, 1H), 4.49–4.45 (m, 2H), 4.41 (q, *J* = 7.0 Hz, 2H), 3.76-3.73 (m, 1H), 3.62–3.59 (m, 1H), 3.41 (s, 3H), 2.64–2.60 (m, 1H), 2.47–2.44 (m, 1H), 1.59–1.56 (m, 1H), 1.50 (s, 3H), 1.39 (t, J =7.0 Hz, 3H), 1.33 (s, 3H). ^13^C-NMR (125 MHz, CDCl_3_) δ 161.4, 159.2, 145.9,111.1, 96.5, 86.8, 84.1, 69.7, 62.4, 55.6, 55.0, 45.6, 33.2 ,27.0, 24.6,14.0; HRMS (ESI) calcd for C_16_H_27_N_2_O_8_ [M+H]^+^ = 375.1767, found 375.1761.

*2-(Hydroxyimino)-N^1^-((3aS,4R,6R,6aR)-6-((methoxymethoxy)methyl)-2,2-dimethyl-tetrahydro-3aH-cyclopenta[d][1,3]dioxol-4-yl)malonamide* (**10**). Compound **9** (0.246 g, 0.66 mmol), dissolved in a saturated solution of ammonia in methanol (15 mL), was stirred for 12 h at 0 °C. After the reaction was complete, the mixture was concentrated under reduced pressure. The residue was purified by silica gel column chromatography using CHCl_3_/CH_3_OH (99:1) to give compound **10** (0.23 g, 99.1%). ^1^H-NMR (500 MHz, CDCl_3_) δ 9.70 (s, 1H), 8.60 (d, *J* = 9.0 Hz, 1H), 6.00 (brs, 1H), 4.78–4.76 (m, 2H), 4.62–4.61 (m, 1H), 4.52–4.49 (m, 1H), 4.42–4.40 (m, 1H), 3.77 (m, 1H), 3.74 (m, 1H), 3.41 (s, 3H), 2.72–2.65 (m, 1H), 2.51–2.48 (m, 1H), 1.60–1.57 (m, 1H), 1.52 (s, 3H), 1.33 (s, 3H). ^13^C-NMR (125 MHz, CDCl_3_) δ 165.1, 162.8, 137.1, 111.8, 96.5, 86.7, 83.7, 68.9, 55.9, 55.4, 44.8, 33.3, 27.1, 24.7; HRMS (ESI) calcd for C_14_H_24_N_3_O_7_ [M+H]^+^ = 346.1614, found 346.1613.

*2-Amino-N^1^-((3aS,4R,6R,6aR)-6-((methoxymethoxy)methyl)-2,2-dimethyltetrahydro-3aH-cyclopenta-[d][1,3]dioxol-4-yl)malonamide* (**11**). A mixture of compound **10** (0.110 g, 0.32 mmol) and 10% Pt/C (20 mg) in methanol (30 mL) was stirred under 14 psi pressure of hydrogen at room temperature for 0.5 h. The catalyst was then filtered off using a bed of celite and the filtrate was evaporated under reduced pressure. The residue was purified by silica gel column chromatography using CHCl_3_/CH_3_OH (97:3) to give **11** (0.082 g, 77.3%). ^1^H-NMR (500 MHz, CDCl_3_) δ 8.5–8.47 (m, 1H), 7.7–7.73 (m, 1H), 5.75 (brs, 1H), 4.75–4.69 (m, 2H), 4.5–4.50 (m, 1H), 4.4–4.34 (m, 2H), 4.16 (brs, 1H), 3.7–3.67 (m, 1H), 3.5–3.55 (m, 1H), 3.40 (s, 3H), 2.57–2.43 (m, 4H), 1.5–1.52 (m, 1H), 1.51 (s, 3H), 1.32 (s, 3H). ^13^C-NMR (125 MHz, CDCl_3_) δ 171.2, 169.4, 111.5, 96.6, 87.0, 83.8, 69.3, 57.0, 55.8, 55.4, 45.2, 33.5, 27.1, 24.7; HRMS (ESI) calcd for C_14_H_26_ N_3_O_6_ [M+H]^+^ = 332.1822, found 332.1801.

*5-Hydroxy-1-((3aS,4R,6R,6aR)-6-((methoxymethoxy)methyl)-2,2-dimethyltetrahydro-3aH-cyclopenta-[d][1,3]dioxol-4-yl)-1H-imidazole-4-carboxamide* (**12**). To the solution of 11 (0.082 g, 0.25 mmol) in anhydrous ethanol (8 mL) was added triethyl orthoformate (8 mL) and acetic acid (0.1 mL) and the mixture was heated at 90 °C under argon for 1.5 h. After the reaction was complete, the solvent was removed under reduced pressure and the residue was purified by HPLC (C_18_ column) to give 12 (0.047 g, 55%). ^1^H-NMR (500 MHz, DMSO-*d_6_*) δ 8.25 (s, 1H), 7.13 (brs, 1H), 6.66 (brs, 1H), 4.89–4.86 (m, 1H), 4.60 (s, 2H), 4.50–4.45 (m, 2H), 3.54–3.51 (m, 2H), 3.28 (s, 3H), 2.29–2.25 (m, 1H), 2.21–2.18 (m, 1H), 2.01–1.96 (m, 1H), 1.46 (s, 3H), 1.24 (s, 3H); ^13^C-NMR ( 125 MHz, CD_3_OD) δ 163.1, 155.7, 125.1, 113.4, 100.1, 96.2, 83.4, 81.6, 68.3, 59.5, 54.2, 43.9, 33.6, 26.4, 24.1; UV λ_max_ (MeOH) 240 nm (ε 4100), 284 nm (ε 7900); HRMS (ESI) calcd for C_15_H_24_N_3_O_6_ [M+H]^+^ = 342.1665, found 342.1667.

*1-((1R,2S,3R,4R)-2,3-Dihydroxy-4-(hydroxymethyl)cyclopentyl)-5-hydroxy-1H-imidazole-4-carbox-amide* (**3**). Compound **12** (37 mg, 0.11 mmol) in methanol (8 mL) and 1N HCl (1 mL) was heated at 55 °C for 1 h. After cooling to room temperature, the reaction mixture was treated with saturated sodium bicarbonate to adjust its pH to 6. The solvent was then removed under reduced pressure and the residue was purified by HPLC using a Delta Pak C_18_ (15 µm, 10 Å) column with the eluting solvent system being water /acetonitrile (acetonitrile from 0 to 100%). The flow rate was 5 mL/min and the retention time was 55.8 min (254 nm detection). After lyophilization, 14.9 mg of pure title compound **3** was obtained (53.6% yield). ^1^H-NMR (500 MHz, D_2_O) δ 8.16 (s, 1H), 4.51 (unresolved dd, 1H), 4.32 (m, 1H), 4.00 (m, 1H), 3.67–3.61 (m, 2H), 2.44–2.38 (m, 1H), 2.23-2.19 (m, 1H), 1.61–1.55 (m, 1H). ^13^C-NMR (125 MHz, CD_3_OD) δ 163.3, 156.2, 125.3, 100.3, 75.2, 72.2, 63.0, 58.3, 45.3, 28.6; UV λ_max_ (MeOH) 240 nm (ε 4,200), 283 nm (ε 8,600); HRMS calcd for C_10_H_16_N_3_O_5_ [M+H]^+^ = 258.1090, found 258.1104. 

### 3.2. Antiviral Activity Assays

The RNA antiviral activity protocols involved the following assays: virus yield reduction, neutral red and visual (http://www.usu.edu/iar/In_vitro_assays.html). 

## 4. Conclusions

Synthesis of compound **3**, the first carbocyclic analog of bredinin, is reported. The methodology developed is general and can be applied to the synthesis of other carbocyclic analogs of this biologically-active natural nucleoside. The antiviral evaluation revealed that the compound shows low activity against some RNA viruses. Further antiviral studies are in progress.
